# Efficiency of occlusal splint therapy on orofacial muscle pain reduction: a systematic review

**DOI:** 10.1186/s12903-023-02897-0

**Published:** 2023-03-28

**Authors:** Sylwia Orzeszek, Marta Waliszewska-Prosol, Dominik Ettlin, Piotr Seweryn, Marcin Straburzynski, Paolo Martelletti, Andrej Jenca, Mieszko Wieckiewicz

**Affiliations:** 1 Department of Experimental Dentistry, Wroclaw Medical University, Wroclaw, Poland; 2 Department of Neurology, Wroclaw Medical University, Wroclaw, Poland; 3 Center of Dental Medicine, University of Zurich, Zurich, Switzerland; 4 Department of Family Medicine and Infectious Diseases, University of Warmia and Mazury, Olsztyn, Poland; 5 Department of Clinical and Molecular Medicine, Sapienza University, Rome, Italy; 6 Clinic of Stomatology and Maxillofacial Surgery, Faculty of Medicine, University Pavol Josef Safarik and Akademia Kosice, Kosice, Slovakia

**Keywords:** Muscle pain, Myofascial pain, Myalgia, Occlusal splint, Occlusal appliance, Temporomandibular disorders, Orofacial pain

## Abstract

**Background:**

This systematic review aims to examine the existing original studies to determine the effectiveness of occlusal splints (OSs) in the management of orofacial myalgia and myofascial pain (MP) in comparison with no treatment or other interventions.

**Materials and methods:**

Based on the inclusion and exclusion criteria of this systematic review, randomized controlled trials were qualified, in which the effectiveness of occlusal splint therapy in the management of muscle pain was examined in comparison with no treatment or other interventions. This systematic review was conducted according to the guidelines of Preferred Reporting Items for Systematic Reviews and Meta-Analysis 2020. The authors searched three databases (PubMed, CINAHL (The Cumulative Index to Nursing and Allied Health Literature) and Scopus) for English publications published between January 1, 2010, and June 1, 2022. The last database search was carried out on June 4, 2022. Data were extracted from the included studies and assessed for risk of bias using the revised Cochrane risk-of-bias tool for randomized trials.

**Results:**

Thirteen studies were identified for inclusion in this review. In total, 589 patients were diagnosed with orofacial muscle pain who underwent education and various forms of therapy including different types of OSs, light emitting diode therapy, acupuncture, low-level laser therapy, device-supported sensorimotor training, Kinesio Taping, myofunctional therapy, and physical therapy. All studies included demonstrated a high risk of bias.

**Conclusions:**

There is insufficient evidence regarding whether OS therapy in the treatment of orofacial myalgia and MP offers an advantage over other forms of interventions or no treatment. Further reliable clinical studies in this area are needed to improve the quality of research, which should be performed with larger groups of blinded respondents and controls.

**Clinical relevance:**

Due to the large-scale nature of orofacial muscle pain, it is assumed that each dental clinician will meet patients with orofacial muscle pain repeatedly in daily practice; hence, the review of the effectiveness of OSs in the management of orofacial myalgia and MP is necessary.

## Introduction

Temporomandibular disorders (TMD) involve temporomandibular joints (TMJs), masticatory muscles, and surrounding structures impairments. In accordance to the Diagnostic Criteria for Temporomandibular Disorders (DC/TMD) TMD could be divided in group I: temporomandibular joint disorders; group II: masticatory muscle disorders; group III: headache, and group IV: associated structures [1]. Depending on the population studied according to the age group and gender, the prevalence of TMD ranges from 5 to 12% [2, 3]. Common symptoms of TMD include TMJ noises, limitations and/or deviations of mandible movements, myalgia, myofascial pain (MP), arthralgia, and headache due to TMD [4, 5] The pathophysiology of TMD is not completely understood. TMD have a multifactorial etiology, and some studies reported that central sensitization may play a role in chronic pain [6]. Vale Braido et al. in their work examined whether the somatosensory functions and symptoms associated with central sensitization (CS) differ in people with painful temporomandibular disorder (TMD) depending on the presence of migraine (MIG) or MIG + headache attributed to TMD (HAT). TMD + MIG + HAT had higher chronic pain intensity (*p* = 0.001), disability points (*p* = 0.045), graded chronic pain scale (*p* = 0.007), and higher somatization (NSPS) scores (*p* = 0.012), compared to the other groups. Their results show the importance of considering the association of primary and secondary headaches when assessing TMD and its implications for the persistence of CS signs and symptoms [7]. The relationship between sleep disorders and temporomandibular disorders in adults has also been studied. Pala Mendes et al. in their systematic review confirmed the relationship between obstructive sleep apnea, insomnia, snoring and gastroesophageal reflux with TMD. These results indicate how many clinical implications for the overall health of patients are associated with the occurrence of temporomandibular disorders [8].

Temporomandibular disorders are the second most common musculoskeletal disorders that causes pain and disability [9]. Myalgia and MP are the most prevalent disease entities among TMD [10]. Masticatory muscle pain is the second most prevalent condition in the orofacial region after toothache. Whereas the most prevalent temporomandibular joint disorders is disc displacement with reduction, approximately 26% in adults/elderly and 7.5% in children/adolescents [8]. Both pain and functional limitation of mandibular mobility impair the everyday activities of patients, resulting in a significant reduction in the quality of life, thereby posing a significant public health problem [4]. Due to the large-scale nature and of masticatory muscle pain, it is assumed that dental clinicians will regularly encounter TMD patients with masticatory muscle pain in daily practice. Hence, effective therapies for the management of myalgia and MP are crucial.

Therapies used in the management of TMD can be classified into noninvasive, minimally invasive, and invasive [11]. The most frequently used and most easily available noninvasive therapies include occlusal splints (OSs), medications, and physical and laser therapy (LST) [4, 11, 12]. Minimally invasive therapies such as dry needling are also applied [12, 13]. OSs are defined as a “removable artificial occlusal surface affecting the relationship of the mandible to the maxillae used for diagnosis or therapy; uses of this device may include, but are not limited to, occlusal stabilization for treatment of temporomandibular disorders, diagnostic overlay prior to extensive intervention, radiation therapy, occlusal positioning, and prevention of wear of the dentition or damage to brittle restorative materials such as dental porcelain” [14]. The following mechanisms of action of oral splints have been reported: muscle relaxation/interference with parafunctional habits or muscle tightening, protection of teeth and jaws, normalizing periodontal ligament proprioception, and alteration of the jaw joint space and redistribution of condylar shear forces [15, 16]. Various types of OSs are used in the management of TMD. The majority of the studies describe the use of the so-called stabilization splints (such as a Michigan splint or centric relational appliance), anterior repositioning splints, and anterior bite splints [17]. Due to the high prevalence of the orofacial muscle pain and the popularity of occlusal splint therapy in daily dental practice and confusing efficiency of the occlusal splint therapy in orofacial muscle pain reduction, the systematic review of available clinical studies related to this issue is justified.

Therefore, this systematic review aimed at examining published randomized controlled trials (RCTs) to determine the effectiveness of OSs in the management of orofacial myalgia and MP in comparison with no treatment or other interventions.

## Methods

### Eligibility criteria

RCTs published between January 1, 2010, and June 1, 2022, in English that compared OS therapy to no treatment (understood as education, such as information about nature of TMD, behavioural change, counselling (CSL), guidance, assurance, self-exercise and self-massage), light emitting diode (LED) therapy, acupuncture, low-level LST, device-supported sensorimotor training, Kinesio Taping (KT), myofunctional therapy, and physical therapy were selected for analysis. This review included adults aged 18 years or older with the presence of myofascial pain in accordance with the Research Diagnostic Criteria for Temporomandibular Disorders (RDC/TMD); myalgia, MP, tendonitis, and myositis in accordance with the Diagnostic Criteria for Temporomandibular Disorders (DC/TMD); or a clear clinical diagnosis including signs and symptoms of orofacial muscle pain [1, 18]. The population, intervention, comparison, outcomes, and study criteria were followed (Table 1).

**Table 1:** The population, intervention, comparison, outcomes, and study—the key components of the research questions

“P” population	Adult patients diagnosed with masticatory muscle pain based on DC/TMD, RDC/TMD, or any other protocol providing clear signs and symptoms of myalgia and/or myofascial pain diagnosis
“I” intervention	Different types of intraoral appliances used in the management of masticatory muscle pain
“C” comparator	No treatment understood as education including information about nature of TMD, behavioural change and self-care, guidance, counseling, assurance, self-exercise and self-massage, conservative therapy including LED therapy, acupuncture, low-level laser therapy, device-supported sensorimotor training, Kinesio taping, myofunctional therapy, and physiotherapy
“O” outcomes	Primary outcome of interest: the effect of intraoral appliances on the severity of masticatory muscle pain Secondary outcome of interest: determine what type of cotherapy used with a splint or as an isolated form of therapy will have the highest impact on reducing pain in patients with muscle pain
“T” time	Follow-up periods for the outcome data, minimum 3 months
“S” study design	RCTs comparing therapy with different types of splints with each other or comparing splint therapy with no treatment or other treatment methods

*DC/TMD* Diagnostic Criteria for Temporomandibular Disorders, *RDC/TMD* Research Diagnostic Criteria for Temporomandibular Disorders, *RCT* randomized controlled trial, LED—light emitting diode, *TMD* temporomandibular disorders

The following studies were excluded: nonrandomized controlled clinical studies, RCTs that examined pain without clearly implicating masticatory muscles, studies from which detailed data could not be extracted, duplicated studies, studies with unclear descriptions to determine pain intensity, studies that included subjects who had received treatment for TMD prior to the study, studies in languages other than English, studies that included nonpainful TMD, studies that included TMD secondary to psychogenic, neurologic, or metabolic disorders, when the pain or the presence of tender muscles was attributable to systematic diseases, and studies in which patients had a recent history of trauma in the face or neck.

For data synthesis, the selected studies were grouped according to the type of alternative protocol used in the study for therapy with occlusal appliance.

### Information sources and search strategy

This systematic review was conducted in accordance with Preferred Reporting Items for Systematic Review and Meta-Analysis 2020 (PRISMA 2020) [19].

A structured electronic search was conducted between March 2022 and June 2022, in which two authors (S.O. and P.S.) independently searched RCTs published in English between January 1, 2010, and June 1, 2022, in PubMed, Scopus, and CINAHL (The Cumulative Index to Nursing and Allied Health Literature) databases using various combinations of the following MeSH terms and keywords: (1) masticatory muscle, (2) temporomandibular disorders, (3) myalgia, (4) myofascial pain, and (5) masticatory muscle pain, with each of the following phrases: (a) appliance, (b) splint, and (c) device. In addition, relevant studies were hand-searched. The last database search was carried out on June 4, 2022.

The selection of articles was carried out in two stages. To cover all potential studies, two authors independently performed a double search. After all articles were identified, a database was generated to organize the publications, remove duplicates, and double-screen the included studies. Two researchers (S.O. and P.S.) independently reviewed the titles and abstracts and qualified the studies for further analysis. In the case of doubts about the inclusion of a particular study, the two researchers discussed them between themselves, and a third researcher was asked to provide an independent opinion (M.W.).

### Selection process

Full-text versions of the eligible studies were obtained and carefully reviewed by two independent reviewers for inclusion (S.O. and P.S.). Disputes and ambiguities were resolved by a third reviewer (M.W.).

### Data collection process

Data were collected using a customized data extraction form that included contents such as the title of the study, author names, duration of the study, year of publication, study population, diagnostic criteria (DC), methods of randomization, groups, types of intervention including the type of occlusal appliance, comparators, characteristics of participants (age and gender), follow-up and times of measurement, outcomes (primary and secondary), comparison of results between groups, and conclusions.

### Data items

The primary outcome is the reduction in pain in the masticatory muscles after occlusal splint therapy. The secondary outcome is to determine what type of co-therapy used with a splint or as an isolated form of therapy will have the highest impact on reducing pain among patients with orofacial muscle pain.

### Risk of bias assessment

Using a Cochrane Collaboration’s tool for assessing the risk of bias in randomized trials, two authors independently assessed the risk of bias of all studies included in this systematic review. In this tool, six positions were used to measure the risk of bias: selection bias (random sequence generation, and allocation concealment), blinding of participants and personnel, blinding of outcome assessment, incomplete outcome data, selective outcome reporting, and other bias. The overall risk of bias of the individual studies was categorized as follows: low, if all domains had a low risk of bias; unclear, if one or more domains had an unclear risk of bias; or high, if one or more domains had a high risk of bias [20].

## Results

### Study selection

**Fig. 1:**
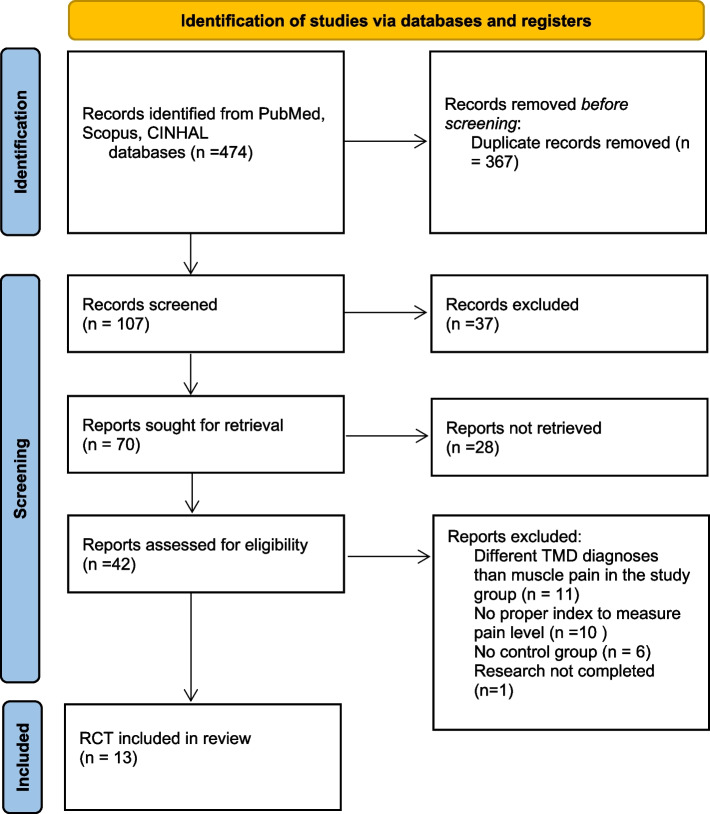
Flowchart of study selection. Abbreviations: TMD, temporomandibular disorders; RCT, randomized controlled trial

The electronic search of the three databases resulted in 474 records. No additional records have been identified in the references of the included studies. After removing duplicates, 107 records remained. Screening of the titles and abstracts of the studies based on the inclusion and exclusion criteria resulted in the removal of further 37 studies. Both authors independently assessed all studies identified in the search. Any disputes regarding qualifying a study to this systematic review or interpretation of the analyzed research were discussed with the third author and resolved. The inclusion criteria were as follows: RCTs in English published between January 1, 2010, and June 1, 2021, the study group being adults, and the study group consisting of at least 20 individuals diagnosed with masticatory muscle pain. The following served as the exclusion criteria: works other than RCTs, published in languages other than English, a study group of less than 20 individuals, and a diagnosis that is not muscular. Manuscripts of the remaining studies were comprehensively assessed for eligibility. Ultimately, 13 articles were included in this systematic review [21–33]. All eligible papers were assessed for the risk of bias. The authors of this review classified all included studies as those with a high risk of bias. During the analysis of full-text versions, some studies were rejected due to different TMD diagnoses than muscle pain in the study group, inclusion of TMD patients without pain, no proper index to measure the pain level, or no control group [34–38]. One study was not completed during the writing of this systematic review [39]. Details on the selection of studies are shown in a flowchart (Fig. 1).

The selection process resulted in RCTs in which therapy with various types of OSs was compared with no treatment, instruction, guidance, CSL, self-exercise or self-massage, and additional forms of therapy used alone or together with OSs: LED therapy, acupuncture, low-level LST, KT, myofunctional therapy, and physical therapy.

### Study characteristics

A total of 589 participants were included: 276 in the OS therapy group, 108 in the conservative and CSL therapy group, 30 in the acupuncture group, 30 in the LST group, 23 in the device-supported sensorimotor training group, 16 in the KT therapy group, 10 in the myofunctional therapy group, 37 in the manual therapy group, and 59 in the LED therapy group. Only one study [26] used DC/TMD as the DC, one used the pressure pain threshold [27], one used sensitivity in the pressure area [29], and the other studies used RDC/TMD. The length of the follow-up ranged from 4 weeks to 6 months. The OSs used could be distinguished as follows: stabilization splints used in 207 patients [21, 22, 24, 25, 27, 30–33], reflex splints used in 59 patients [22, 25, 28], and repositioning splints used in 10 patients [29]. Only one study relied on the characteristic pain intensity (CPI) scale to assess the intensity of pain, whereas the remaining studies used the visual analog scale (VAS) to assess pain. Characteristics of the included studies are presented in Table 2.

**Table 2:** Characteristics of the included studies

Reference	First author	Publication year	Study population	Mean age	Sex	Total sample size	Diagnostic criteria	TMD qualification	Groups	Types of intervention including types of occlusal appliance
14	Ficnar T	2013	63	34,66	79,4% women, 20,6% men	63	RDC/TMD	Ia/Ib myofascial pain	Group 1 (CO)—conservative therapy Group 2 (SS)—conservative therapy and a laboratory-made stabilization splint Group 3 (SB)—conservative therapy and the SOLUBrux splint	Stabilization splint, SOLUBrux splint
8	Costa D	2021		31,4	80% women, 20% men	70	RDC/TMD	Ia/ib Myofascial TMD	Group 1 (G1) only conventional therapy with OS; Group 2 (G2) treatment with OS and therapy with LED (device turned off) Group 3 (G3) LED therapy (infrared) Group 4 (G4) LED therapy (infrared) Group 5 (G5) OS associated with LED (infrared) therapy; Group 6 (G6) OS therapy plus infrared LED	Stabilizing plates, used during sleep for 4 weeks
15	Conti P	2012		I 38,09, 1135,25 III38,14	I women 80,9%, II women 87,5%, III 100% women	51	RDC/TMD	Myofascial	Group I stabilization occlusal splint and counseling Group II anterior device nociceptive trigeminal inhibitory (NTI) system, counseling Group III only counseling (the control group)	Full coverage acrylic stabilization occlusal splint, anterior device nociceptive trigeminal inhibitory (NTI)
16	Grillo C	2014		18–45	Women using contraceptives	40	RDC/TMD	Myogenic dysfunction	Group 1—acupuncture group Group 2—splint group	Flat occlusal plane appliance (control 1 session per week, final evaluation after 4 weeks)
17	Oz. S	2010		32,84	34 women, 6 men	40	RDC/TMD	Myofascial	Group 1—study group; low-level laser Group 2—control group; occlusal splints	Stabilization occlusal splint described by Okeson wearing 24 h/d for 3 months
18	Giannakopoulos	2018	45	18–45	Female	45	RDC/TMD	Myofascial	A. sensorimotor training B. occlusal splint	Conventional Michigan splint, use during sleep only
19	Manfredini D	2017	30 patients	35,3 + -9.4	Female	30	DC/TMD	Myofascial pain	LST (laser therapy), OA (oral appliance therapy), CSL (counseling)	Flat occlusal appliance covering the maxillary teeth at night for 3 weeks and then intermittent use for the following two months
20	Keskinruzgar A	2018	34 patients with sleep bruxism	Kinesio group 27.38 + -9.05. 26.11 +—8.71	Mixed	34	Pressure pain threshold for masseter and temporal muscle, mouth opening, VAS	Tenderness on palpation, teeth abrasions, teeth grinding reported by bedtime friend hypertrophy of masseter muscle	Kinesio taping group—16 occlusal splint—18	Occlusal appliance made according to Okeson models. 0,5 mm thick thermoplastic hard splint
21	HAsanoglu Erbasar	2017	40 patients with myofascial pain		Mixed		RDC/TMD	RDC/TMD group I	Group 1—guidance, counseling, assurance, behavioral changes advice, Group 2—NTI-tss device	NTI-tss device
22	Vicente-Barrero M	2012	20 patients	18–58	17 females, 3 males	20	Sensitivity to pressure on area: preauricular, masseter muscle, temporal muscle, trapezius	Muscle pain during palpation	1. acupuncture group 2. occlusal splint group	Decompression splints with canine guidance
23	Claudia Maria de Felicio	2014	40	13–68	Female	22	RDC/TMD, Helkimo index	Myofascial	T group—OMT OS group—occlusal splint, SC—symptomatic control group with TMDs AS—asymptomatic control group	Occlusal splint Michigan type
24	Robert van Grootel	2017	72 patients	1 group 31,4 2 group 29,0	1 group- 95% female; 2 group 91% female	72	RDC/TMD	Group Ia and Ib	1 group—physiotherapy, 2 group—splint therapy	Michigan splint
25	Michelotti A	2012	44 patients	2 group 31,4 1 group- 31,1	Mixed	41	RDC/TMD	Ia group or Ib group	1. Occlusal splint group 2. Control—information about TMDs and self-care measures	Michigan splint (only during sleep)

Reference	Types of comparator	Blinding	Allocation sequence	Follow-up	Losses to follow-up	Primary outcome	Secondary outcome	Results	Conclusion
14	Conservative therapy	Not described	Via randomization	3 months	2 Patients from CO, 3 patients from SS	Pressure-sensitive areas upon muscle palpation	Extent of vertical movement (incisal edge distance in mm)	Not established significant differences regarding pain reduction (muscular/joint pain) and mouth opening between the various therapeutic approaches; TMDs should initially be treated with conservative therapy consisting of self-exercises, as well as drug-based and manual treatment	In the SS and SB groups, a statistically significant improvement in mouth opening, especially in the case of the initial limitation of this range
8	LED therapy	Only the irradiation was hidden	Randomized	4 weeks of treatment, last evaluation 30 days after finishing treatment	11	Pain intensity (VAS)	Muscle activity (electromyography) Blood lactate level	Pain intensity significantly decreased both post-therapy and 30 days post-treatment	The combination of LED therapy and occlusal splint achieves superior results compared to isolated treatments, and the protocol of two sessions per week proved to be better
15	Counseling	No information	Via randomization	2 weeks, 6 weeks, 3 months	12	Pain intensity, pressure pain threshold (PPT) of the masticatory muscles	Patients who halved their VAS (visual analog scale)	All of the management strategies used in the present study provided a significant improvement in the pain levels of myofascial pain when judged by the VAS	The simultaneous use of occlusal devices appears to produce an earlier improvement high percentage of patients responsive to the treatment in groups I and II, which include those who had decreased their VAS Values by at least 50% (28), highlighting the importance of the occlusal splint in the management of myogenic pain
16	Acupuncture (traditional Chinese medicine) 4 sessions (one per week, 20 min duration)	No information	Via randomization	4 weeks	4	Pain intensity—VAS, pain pressure threshold (PPT)	Range of mouth opening (RMO), electromyographic activity	Reduction in pain in each group. Increase in RMO in both groups	Both strategies can be used equally for control of chronic pain related to TMDs
17	Low-level laser therapy (applied 2 times per week- 10 sessions)	Double blind	Randomly divided	90 days	No information	Self-report pain—VAS. Pressure pain threshold	Mandibular movement	Both groups—statistically significant improvement in vertical movements after treatment In both groups, tenderness to muscle palpation and PPT evaluations decreased significantly	Low-level laser therapy is effective like occlusal splining pain release and mandibular improvement in myofascial pain
18	Device-supported sensorimotor training (a prefabricated device with liquid-filled elastic pads)	No information	Via randomization	6 weeks, 12 weeks	3 patients from group A	Pain intensity (current, average, worst pain) NRS, characteristic pain intensity (CPI)	EMG activity, bite—force (bite force device)	Significant pain reduction in both groups EMG activity was not significantly different in both groups after treatment	Device-supported training could be a cost-effective alternative (or additional) treatment for myofascial functional pain TMD patients
19	Laser therapy, counseling	TMD practitioner who assessed outcome variables was blinded to patient groups assignment	Randomized	6 months	1	Visual analog scale (VAS) pain levels	Muscular index (MI) of the Craniomandibular Index	After 3 weeks VAS values decreased significantly only in the LST group After six months for VAS values, positive changes were still shown for LST and were also shown for the appliance therapy and CSL groups	All three treatment groups improved at six months. The difference in the short-term effectiveness of LST and OA, with respect to CSL alone, may suggest that active treatments should be directed to maximize the positive changes in the short-term period
20	Occlusal appliance, Kinesio taping	Not described		5 weeks		Significantly lower VAS scores in both splint group and Kinesio taping group	Significantly increased mouth opening in both groups	Significant decrease in VAS values, increase in muscle pain threshold in masseter and temporal muscles in both groups	Kinesio taping is easy and reliable treatment, which can be applied to patients with bruxism
21	Guidance, counseling, assurance, behavioral changes	Evaluation and data collection were performed by another clinician who was unaware of patient group	Randomized			Pain reduction VAS	Jaw function	Reduction in pain with time was observed; no significant difference regarding pain reduction was noted between the groups. Jaw function gradually improved in both groups, no significant difference between the groups (*P* = 0,927)	Integration of NTI-tss device into protocol of counseling, guidance, and assurance did not provide any additional benefit for patients
22	Acupuncture therapy			5 weeks	0	VAS, pain upon palpation of masticatory muscles	Measurements of mouth opening and jaw lateral deviation	Both groups of patients showed reduction in myofascial pain in the short term	Acupuncture is an effective complement and/or an acceptable alternative to decompression splints in the treatment of myofascial pain and temporomandibular joint pain dysfunction syndrome
23	Orofacial Myofunctional Therapy	No blinding	GraphPad software	120 days		Helkimo index	Questionnaire about the severity of their signs and symptoms	Group treated with OMT had significantly lower pain after treatment in comparison with SC (symptomatic control group), splint therapy group also revealed significant improvement, with some advantages for orofacial myofunctional therapy group	OMT has positive effects on patients with TMDs such as reduction in pain sensitivity on palpation of all muscles of the stomatognathic system
24	Physiotherapy	Blinded assessor after treatment		After 6 months and again after 6 more months		Clinical examination—pain intensity during jaw movements, palpation of jaw muscles and during clenching	Anamnestic questionnaire	Success rate for physiotherapy was similar to that of splint therapy in short term and long term. The duration of physiotherapy is on average 10.4 weeks shorter than that of splint therapy, so it might be the first step for patients without severe active sleep bruxism or psychological problems	Physiotherapy and splint therapy have similar success rates and effectiveness
25	Group with only information about TMD		Randomized	3 months	3	VAS scores for spontaneous muscle pain	Pain-free maximal mouth opening, headache, pain during chewing	Changes in spontaneous muscle pain differed significantly between the groups. Changes did not differ significantly between groups pain-free maximal mouth opening (*P* = .528; effect size = 0.20); headache and pain on chewing (*P* ≥ .550, effect size ≤ 0.10)	During short period of time, education was slightly more effective than an occlusal splint in treating spontaneous muscle pain

*RDC/TMD* Research Diagnostic for Temporomandibular Disorders, *OMT* orofacial myofunction therapy, *OS* occlusal splint, *TMD* temporomandibular disorders, *VAS* visual analog scale, *DC/TMD* Diagnostic Criteria for Temporomandibular Disorders, *NRS* numeric rating scale

### Risk of bias in studies

Overall, all 13 studies were identified as being at a high risk of bias as they showed a high risk of bias in at least one of the seven factors analyzed.

In terms of random sequence generation, eight studies were assessed as having an “unclear risk of bias” because of insufficient information about randomization methods. Five studies adequately described the approach of allocating subjects to groups. With respect to the next factor—concealment of information about group allocation—no study properly provided information, and thus, all studies were categorized as having an “unclear risk of bias.” In the next domain assessed (blinding of participants and personnel), all studies had a high risk of bias because patients cannot be blinded in the case of OS therapy versus other therapies.

Regarding the detection bias, six studies were considered at low risk. In this section, not only the blinding of assessors was considered important, but also the type of evaluation method of patients. Two studies were graded as having high risk because the same examiner assessed the patients and was not blinded to the patient group assignment. Five studies showed an unclear risk of bias due to a lack of information about the blinding of the examiner. The remaining studies were graded as having a low risk of bias.

Only one study showed a high risk of bias due to incomplete data (attrition bias), which was attributable to the high number of dropouts. Unclear risk of bias was observed in five studies and was related to insufficient information about the number of subjects lost during the study. The rest of the studies were considered as having a low risk of bias as they had precise information on the number of dropouts and carried out statistical analysis using only data from patients who completed the study.

Selective reporting was observed in only one study. Three studies showed a high risk of bias due to other sources of bias: due to important differences in the study group, poor assessment methods, and poor data reporting, respectively.

Simplified information regarding the assessment of the risk of bias is provided in Table 3.

**Table 3:** Risk of bias assessment

	*Random sequence generation*	*Allocation concealment*	*Blinding of participants and personnel*	*Blinding of outcome assessment*	*Incomplete outcome data*	*Selective reporting*	*Other bias*
*Ficnar, 2013 *[21]	**?**	**?**	** − **	**?**	** + **	** + **	** + **
*Costa, 2021 *[13]	** + **	**?**	** − **	** + **	** + **	** + **	** + **
*Conti, 2012 *[22]	**?**	**?**	** − **	** + **	** − **	** − **	** + **
*Grillo, 2015* [23]	**?**	**?**	** − **	** − **	**?**	** + **	** − **
*Giannakopoulos, 2018* [25]	**?**	**?**	** − **	**?**	** + **	** + **	** + **
*Manfredini, 2018 *[26]	** + **	**?**	** − **	** + **	** + **	** + **	** + **
*Keskinruzgar, 2019 *[27]	**?**	**?**	** − **	**?**	**?**	** + **	** + **
*Erbasar, 2017* [28]	**?**	**?**	** − **	** − **	**?**	** + **	** − **
*Oz, 2010* [24]	**?**	**?**	** − **	** + **	** + **	** + **	** + **
*Vicente-Barrero, 2012* [29]	**?**	**?**	** − **	**?**	**?**	** + **	** − **
*de Felicio, 2010* [30]	** + **	**?**	** − **	**?**	**?**	** + **	** + **
*Grootel R, 2017* [31]	** + **	**?**	** − **	** + **	** + **	** + **	** + **
*Michelotti. 2012* [32]	** + **	**?**	** − **	** + **	** + **	** + **	** + **

** + **low risk of bias, **?** unclear risk of bias − high risk of bias

### Results of individual studies

Thirteen studies from the analyzed literature met the inclusion criteria and were assessed using primary and secondary outcomes. The results of each included study are summarized below:

#### OSs versus acupuncture

Grillo et al. compared the effects of acupuncture with those of flat occlusal plane appliance. They evaluated 40 women with myogenic temporomandibular dysfunction, who were randomly divided into two groups: acupuncture and OS. The effect of 4 weeks of treatments on masseter and anterior temporal muscles was evaluated using electromyographic (EMG) activity and pain pressure threshold. Pain intensity was measured using the VAS, and the range of mouth opening was evaluated using a millimeter ruler. All evaluations were conducted at the beginning and the end of the treatment. In both groups, the VAS score was equally reduced (*p* < 0.001) and the increase in the range of mouth opening was significant. Therefore, the authors reported that both therapies can be used with equal effectiveness in the control of chronic pain associated with TMD [23].

Barrero et al. studied the effect of acupuncture in comparison with occlusal decompression splints in TMD patients with muscle pain. They conducted an RCT involving 20 patients who underwent the abovementioned treatment methods. Outcomes were assessed using a visual analog pain scale, the range of mouth opening, lateral deviation of the mandible in millimeters, and assessment of sensitivity to pressure at various points: preauricular, masseter, temporal, and trapezius muscles. Parameters were assessed before and 30 days after the 5-week treatment. Both groups of patients showed a reduction in MP in the short term. Patients treated with decompression splints showed a reduction in subjective pain and pain on pressure points located on the temporal, masseter, and trapezius muscles, but without statistically significant differences (*p* > 0.05). Patients treated with acupuncture experienced significant improvements in all studied parameters (reduced subjective pain, stronger algometer pressure needed to produce pain, improved mouth opening). Pain reduction was statistically significant (*p* < 0.05) for all but one evaluated point: the one located on the masseter muscle (*p* = 0.068). Acupuncture is an effective complement and/or an acceptable alternative to decompression splints in the treatment of MP. The authors did not conduct a long-term follow-up of patients, so these results are limited to the immediate effects of both procedures used. Due to the lack of long-term observations, conclusions regarding the long-term impact of acupuncture could not be made, which is highly important in patients with chronic TMD pain [29].

#### OSs versus LST

Oz et al. compared the effects of low-level LST with those of OSs in patients with the signs and symptoms of MP dysfunction syndrome. A total of 40 patients (34 women and 6 men) were randomly divided into two groups: study group (*n* = 20) who underwent low-level LST (twice per week, for a total of 10 sessions) and control group (*n* = 20) who were instructed to wear OSs 24 h/d for 3 months. The authors presented the conclusions of this study in the form of vertical movements, which showed statistically significant improvements after the treatments in both groups (*p* < 0.01); however, when the groups were compared with each other, no significant differences were observed. In both groups, tenderness to palpation of the muscles decreased significantly. In addition, pressure pain threshold evaluations and VAS scores revealed similar results. This particular type of low-level LST (820 nm, 3 J/cm^2^, 300-mW output power) was shown to be as effective as OSs in pain release and mandibular movement improvements in patients with MP [24].

Manfredini et al. compared three treatment modalities for the management of MP in jaw muscles: LST, oral appliance (OA), and only CSL. VAS pain levels and the muscular index (MI) of the Craniomandibular Index were assessed at baseline, 3 weeks, 3 months, and 6 months. After 3 weeks, VAS values decreased significantly only in the LST group (*p* = 0.018), and significant improvements were observed in the MI (LST, *p* = 0,038; OA, *p* = 0.008). After 6 months, positive changes in VAS values were observed in the LST group (*p* = 0.001) and also shown in the OA (*p* = 0.002) and CSL groups (*p* = 0.048), and improvement in the MI was maintained in both LST (*p* = 0.025) and OA groups (*p* = 0.001). Therefore, the authors of this study concluded that active forms of therapy such as splint therapy and LST support should be used to maximize the positive effect of therapy on pain relief in the short term. However, in the long term, i.e., after 6 months, all forms of therapy had a similar effect [26].

#### OSs versus other appliances

Giannakopoulos et al. compared the short-term therapeutic efficacy of device-supported sensorimotor training with that of standard splint therapy in patients with myofascial TMD pain over a treatment period of 3 months. A total of 45 patients with myofascial TMD pain (graded chronic pain status, I and II) were randomly assigned to two treatment groups (sensorimotor training and conventional splint treatment). The patients were evaluated four times (initial examination and 2, 6, and 12 weeks later) by RDC/TMD, and their EMG activity of the temporal and masseter muscles was recorded during the initial session and after 3 months under conditions of force-controlled submaximum and maximum intercuspation. Significant (*p* < 0.001) pain reduction (sensorimotor training 53% and splint therapy 40%) was achieved in both groups, with no significant differences (*p* > 0.05) between them. EMG activity under submaximum bite force was not significantly different between the first and last sessions (*p* > 0.05). However, during maximum biting, EMG activity was approximately 20% higher (*p* < 0.01) for masseter muscles in both groups after the treatment period. In contrast, a significant increase (*p* < 0.01) for temporal muscles was observed only in the sensorimotor training group. Moreover, sensorimotor training was significantly (*p* < 0.05) more difficult to use than splints, which, according to the authors, is related to the patients’ preference for passive forms of treatment that do not require their involvement. The limitation of this study is the lack of a true control group, i.e., untreated patients with TMD pain, and inability to control the timing of intervention, especially during home exercises. Device-supported sensorimotor training could be a cost-effective alternative (or additional) treatment for functional TMD pain [25].

#### OS versus LED therapy

Costa et al. compared the effects of LED therapy associated with OS on the signs and symptoms of TMDs. They assessed procedures with LED therapy and OSs in six groups of patients: group 1 (G1) was the control and received only conventional therapy with OS; group 2 (G2) was the placebo and received treatment with OS and therapy with LED (device turned off); group 3 (G3) received LED therapy (infrared) once a week; group 4 (G4) received LED therapy (infrared) twice a week; group 5 (G5) received OSs associated with LED (infrared) therapy (once a week); and group 6 (G6) received OS therapy plus infrared LED (two sessions per week). The patients were evaluated before and 30 days after treatment using pain intensity in the masticatory system during all controls. EMG signals of masseter and temporal muscles and blood lactate levels were evaluated before and after the treatment. Groups subjected to combined treatment with splint therapy and LED therapy showed significant differences (*p* < 0.05) from the control group in the analysis of pain intensity and decreased RMS (root-mean-square) values (EMG analysis). The combination of LED therapy and OSs shows superior results than isolated treatments, and the protocol of two sessions per week was associated with a significant reduction (*p* < 0.05) in blood lactate levels [33].

#### OSs versus physical therapy

Felicio et al. studied the effects of orofacial muscle activity therapy (OMT) in the treatment of patients with concomitant painful joints and TMDs, who were randomized into four groups: 10 patients were treated with OMT (group T), 10 were treated with an OS (OS group), and 10 formed the untreated TMD control group (SC). Ten patients without TMDs formed the asymptomatic group (AC). During initial studies, no significant differences were observed between the OMT group, OS group, and control TMD group (*p* > 0.05). After 120 days, better results were observed in the OMT group than in the OS group. Between groups, significant differences were observed in headache frequency (*p* < 0.05), and all groups showed a decreased sensitivity to pain during palpation of all tested muscles, except for TMJs; increased measures of jaw range of motion; decreased Helkimo Index; reduced frequency; and severity of signs and symptoms. The control group did not differ over time in comparison with interventions [30].

Keskinruzgar et al. evaluated the effectiveness of KT in patients with muscle MP and patients with sleep bruxism (SB). They tried to answer the question of whether KT may be an alternative to OSs in the treatment of muscle pain in patients with SB. In this study, 16 patients were treated with KT (Kinesio group), and 18 patients were treated with OSs (splint group,(Sp-Tx). Masseter and temporal muscle pain thresholds (MPPT and TPPT), VAS, and mouth opening values were compared before treatment and at weeks 1 and 5 of treatment. Both KT and OS therapy were significantly associated with reduced muscle pain thresholds, lower VAS values, and increased range of mouth opening. There was no statistically significant difference between the KT and Sp-Tx groups in MPPT, TPPT, VAS, and mouth opening values before and after treatment. KT is an easy-to-use treatment method and has been found to reduce muscle pain and increase the range of mouth opening. It is at least as effective as OS in the treatment of muscle-related pain in patients with SB [27].

Grootel et al. conducted a study on 72 patients, who were randomly assigned to either the physical therapy group (Ph-Tx) or the Sp-Tx group. Following treatment and a 1-year follow-up, the yielding success rate (SR), effectiveness (mean index treatment duration control (TDC)), and Cohen’s d were used as treatment outcomes to determine pain intensity. SR and effectiveness were similar for Ph-Tx and Sp-Tx groups (long-term SR: 51–60%; TDC: − 0.512 to − 0.575). Cohen’s d was 0.86 (Ph-Tx) and 1.39 (Sp-Tx). Treatment duration was shorter in the Ph-Tx group (on average 10.4 weeks less; *p* < 0.001). The Sp-Tx group needed 7.1 visits less (*p* < 0.001). Grootel et al. reported that physical therapy may be preferred as initial therapy over OS therapy in stepped care of myogenous TMDs. With a similar SR and effectiveness, physiotherapy required a shorter duration. Thus, patients whose initial physical therapy was unsuccessful can continue with the subsequent treatment. This stepped-care model reinforces the conclusion on therapy preference as the overall SR hardly depends on the therapy sequence [29].

#### OSs versus education

Michelotti et al. carried out a clinical trial to compare the effectiveness of an education program with that of an OS in treating MP of jaw muscles across a short period. A total of 44 patients were randomly divided into two groups. One group (education group) received information regarding the nature of TMD and self-care measures, whereas another group received an OS. After 3 months, changes in spontaneous muscle pain differed significantly between the two groups (*P* = 0.034; effect size = 0.33). The reduction in pain level is better in the education group. No significant differences in changes in pain-free maximal mouth opening were observed between the two groups (*P* = 0.528; effect size = 0.20). Furthermore, changes in headache and pain on chewing did not differ significantly between the two groups (*P* ≥ 0.550, effect size ≤ 0.10). According to the authors, self-care education as well as extensive communication between the patient and the doctor may be more effective than OSs in the treatment of MP. However, studies on the two protocols used did not report their long-term effects [32].

Erbasar et al. compared the clinical nociceptive trigeminal inhibition–tension suppression system (NTI-tss) device with first-line therapy of MP, which includes guidance, assurance, CSL, and behavioral changes. This trial included 40 patients diagnosed with MP, who were randomly divided into two groups: one group consisted of patients who received guidance, ensuring, CSL, and behavioral change; in the second group, the use of the NTI device was added to the CSL. Patients from both groups experienced a reduction in pain with time; however, no significant differences in pain reduction were observed between the groups (*P* = 0.922). Jaw function gradually improved in both groups, but without significant differences between the groups (*P* = 0.927). The authors concluded that the integration of the NTI-tss device into the therapy protocol did not provide any additional benefit in relieving the symptoms of MP [28].

Conti et al. compared the effectiveness of therapy in the management of pain in masticatory muscles. A total of 51 patients diagnosed with masticatory MP were randomly divided into three groups. Group I received a full-coverage acrylic stabilization OS and CSL, group II received an anterior device NTI system and CSL, whereas group III received only CSL for behavioral changes and self-care without the support of additional intraoral appliances. Behavioral changes were effective in the treatment of MP. However, concurrent use of occlusion devices, especially a stabilization splint, seemed to cause earlier improvement. The authors concluded that the integration of NTI-tss in the treatment protocol provided no added benefit in alleviating MP. It should be noted that the follow-up period is relatively short and the study group is small, which undoubtedly creates the need for further research from this perspective [22].

Ficnar et al. conducted their research on 63 patients diagnosed with myofascial TMDs. The first group (CO group- control group) received conservative therapy, including the use of self-exercises (muscle exercise form according to Prof. Schulte, self-massage techniques, and mouth opening exercises), medication-based therapy using nonsteroidal anti-inflammatory drugs, muscle relaxants, as well as manual therapy. The second group (SS- laboratory-made occlusal appliance group) received a laboratory-made stabilization splint in addition to conservative therapy. The third group (SB) received conservative therapy and the SOLUBrux splint-prefabricated semifinished occlusal appliance. In all three groups, the authors observed a reduction in the pressure-sensitive areas upon palpation of the muscle and TMJs as well as an increase in the extent of vertical movement. With respect to the extent of pain-free active vertical movement of the lower jaw, no statistically significant differences were observed within the CO group, whereas differences were observed between the initial findings and the final examination after 2.5 months in the SS group (*P* = 0.041). However, in the SB group, differences were observed both between the initial findings and the two-weekly follow-up examination (*p* = 0.004) and between the initial findings and the final examination (*p* = 0.021). In the SB group, the increase in active mouth opening accompanied by pain between the initial findings and the 2-weekly follow-up examination was significantly higher (*p* = 0.025). Regarding the overall number of extraoral muscle palpation areas, a reduction in the pressure-sensitive areas was observed in all three groups. These results suggest that TMDs should initially be treated with conservative therapy consisting of self-exercises as well as drug-based and manual treatment. However, a higher improvement in mouth opening was achieved in the SB and SS groups. It should be noted that this effect appeared faster in the group of patients using the semifinished SOLU-Brux occlusal appliance than in the group of patients using the laboratory-made stabilization splint. The authors concluded that semifinished appliances are not only more quickly available but also cheaper, so they should be used as the first-choice method, especially in patients with restricted mouth opening [21].

## Discussion

The authors of this systematic review analyzed RCTs on the effectiveness of various methods used in the management of orofacial pain of myogenic and myofascial origin. To the best of our knowledge, the systematic reviews conducted so far on the effectiveness of splint therapy used in TMD have not focused exclusively on patients with pain [2–4, 40–43]. Due to the wide range of symptoms and dysfunctions included in different forms of TMD, it appears beneficial to separate dysfunctions of muscular origin from pathologies related to the articular disk and degenerative changes in TMJs. Muscle pain is the most common form of TMD [44, 45]. Hence, reliable analyses of the available forms of therapy are crucial in the management of this condition.

The studies included in this review allowed us to compare the effectiveness of OS therapy with other forms such as acupuncture therapy, LST, using other devices than OSs, LED therapy, physiotherapy, and patient education and CSL.

The key findings and considerations of this systematic review are the following: 1. OS therapy showed similar effects as acupuncture, so both therapies can be used with equal effectiveness. However, these studies reported only short-term observations, so data on how long the effects of therapy will remain at the same level were not available; 2. LST is as effective as OS therapy in pain relief and mandibular movement improvement in patients with MP; 3. there are some indications that OS therapy or LST can be used to increase the positive effect of therapy on pain relief in the short term, and long-term observations for these forms of therapy showed an effect similar to that of patients who received CSL and education; 4. device-supported sensorimotor training could be a cost-effective alternative (or additional) treatment for functional TMD pain; 5. the combination of LED therapy and OS therapy achieved superior results compared with individual treatments; 6. physical therapy used in patients with pain of muscular origin resulted in similar effects as splint therapy. However, according to the authors of the included studies, physical therapy reduces the pain of muscular origin in TMD patients faster, and thus, it can be included in the treatment protocol, because if the obtained effect is not sufficient, it can continue earlier with subsequent treatment; 7. CSL for behavioral changes and self-care caused similar effects as splint therapy, so it can be successfully used as the first-choice approach in patients with orofacial muscle pain.

Several studies have analyzed the available treatment methods for patients with various forms of TMD. The conclusions of the present review are similar to theirs. However, there may be some differences as previous works focused on a mixture of patients experiencing myalgia, TMJ degenerative changes, and/or disk pathology. Moraissi et al., in their systematic review with meta-analysis, concluded that for the management of TMD, the best effects were achieved using a repositioning splint and CSL therapy in combination with a hard stabilization splint [17]. Low-quality evidence suggests that a hard stabilizing splint alone is effective in reducing the signs and symptoms of myogenic TMD compared with no treatment (untreated controls) or treatment with non-OSs [17]. Furthermore, other authors described low-quality evidence suggesting that a hard stabilization splint combined with CSL therapy has comparable effectiveness in pain relief and pain severity to that of a hard stabilization splint alone [46–48]. However, some studies indicated that a hard stabilization splint combined with CSL therapy can provide additional benefits over a hard stabilization splint alone [17]. Moreover, some of the previous studies concluded that the hard stabilization splint and the soft stabilization splint yield similar results in subjective pain scores [49, 50]. Hang et al. reported that the effectiveness of OS therapy and exercise therapy is equivalent in pain relief and improvement of mandibular movement in painful TMD patients [4]. However, the aim of the systematic review and meta-analysis proposed by Ferillo et all. was to evaluate the effectiveness of conservative interventions in relieving pain in patients with intracapsular temporomandibular disorders (TMD). According to the authors of this paper, the use of splints and laser therapy is a significantly effective method of pain relief in patients with intracapsular TMD. At the same time, paying attention to the importance of correct diagnosis, because it is largely related to the success of the implemented therapeutic methods [51].

## Limitations at the study level

All included studies were RCTs exclusively in English and had a high risk of bias due to a lack of blinding. Pain was reported in different ways and at different times, and the authors of the studies used different statistical methods, which reduced the number of studies that could be combined to perform a meta-analysis. Furthermore, the study groups were not characterized in terms of pain intensity at the study beginning, which may also affect the results. Moreover, no studies examined the effects of the applied therapy on the level of pain experienced by patients with TMD in the long term. In addition, there were no studies conducted on large groups with an equal gender distribution.

## Conclusions

This systematic review revealed that data to draw definitive conclusions on the effectiveness of OSs for the treatment of orofacial myalgia and MP were insufficient, compared with no treatment or other intervention. Due to the high risk of bias in the research conducted so far, long-term, high-quality blinded research with and large sample sizes is needed.

## Recommendations for future research

Pain in general is a subjective experience perceived variably by different people, reflected by unique activation patterns within the whole brain and the brain regions processing nociceptive stimuli [52]. The experience of masticatory myalgia is reported to be influenced by socioeconomic and other stressful burdens, psychological coping strategies, and biological vulnerabilities [53]. Differences in pain perception are further attributable to patient characteristics such as gender, anxiety, fear of pain, mood states, personality disorders, beliefs, sleep quality, attention, empathy, etc., all of which additionally shape the pain experiences specific to each individual and contribute to timely fluctuations of pain intensity [54, 55]. Taking all these aspects into account, clinical research on masticatory myalgia is an extremely difficult challenge. Furthermore, from a methodological point of view, the choices of instruments measuring all the above parameters remain critical in clinical pain research to adequately assess interindividual comparability and intraindividual reproducibility of both the spontaneous pain experience and the reproducibility of pain intensity rated upon physical stimulation (i.e., psychophysics related to muscle palpation) [56]. In addition, other limitations need to be overcome in relation to the intersession reliability of instruments measuring clinical pain. For instance, in the assessment of clinical pain, patients are not routinely trained for constant interpretations of pain scale parameters across time periods. Finally, in the future, studies should be designed with clear inclusion and exclusion criteria, clarity in the diagnostic process of participants, adequately sized study and control groups, longer observation periods, and blinding. The use of the same methods in the statistical analysis makes it possible to design a study in the form of a meta-analysis of all available methods of therapy for patients with orofacial myalgia and MP.

## Data Availability

The datasets used and/or analysed during the current study available from the corresponding author on reasonable request.

## Abbreviations

TMD: Temporomandibular disorders
RCT: Randomized controlled trial
CINAHL: The Cumulative Index to Nursing and Allied Health Literature
LED: Light emitting diode
TMJ: Temporomandibular joint
RDC/TMD: Research Diagnostic Criteria for Temporomandibular Disorders
DC/TMD: Diagnostic Criteria for Temporomandibular Disorders
MP: Myofascial pain
NSPS: Somatization scores
CS: Central sensitization
MIG: Migraine
HAT: Headache attributed to TMD
LST: Laser therapy
OA: Oral appliance
OS: Occlusal splint
CSL: Counselling
RMS: Root-mean-square values
AC: Asymptomatic group
VAS: Visual analog scale
MI: Muscular index
KT: Kinesio Taping
SB: Sleep bruxism
MPPT, TPPT: Masseter and temporal muscle pain thresholds
Ph-Tx: Physical therapy group
Sp-Tx: Splint group
SR: Success rate
CO: Control group
SS: Laboratory-made occlusal appliance group
SOLUBrux splint: Prefabricated semifinished occlusal appliance
NTI: Anterior device nociceptive trigeminal inhibitory
NSAID: Nonsteroidal anti-inflammatory drug
DC: Diagnostic criteria
NRS: Numeric Rating Scale
CPI: Characteristic pain intensity
